# Application Research on Nb Microalloying of High-Carbon Pearlite Bridge Cable Wire Rods

**DOI:** 10.3390/ma16062160

**Published:** 2023-03-08

**Authors:** Xiaoxiong Zhu, Jie Zhou, Chengyang Hu, Kaiming Wu, Yifu Shen, Yongqing Zhang, Yuedong Jiang

**Affiliations:** 1 College of Materials Science and Technology, Nanjing University of Aeronautics and Astronautics (NUAA), Nanjing 211106, China; 2 Jiangsu Tokyo Rope Co., Ltd., Jiangyin 214445, China; 3 College of Intelligent Manufacturing, Jianghan University, Wuhan 430080, China; 4 Collaborative Innovation Center for Advanced Steels, Wuhan University of Science and Technology, Wuhan 430081, China; 5 Citic Metal Co., Ltd., Beijing 100004, China; 6 Jiangsu Metallurgical Technology Research Institution, Zhangjiagang 215625, China

**Keywords:** Nb microalloying, interlamellar spacing, original austenite, grain size, bridge cable wire rod

## Abstract

The application of Nb microalloying to high-carbon pearlite bridge cable wire rod steel has always been controversial, especially in the actual production process, which will be affected by the cooling rate, holding temperature and final bonding temperature. In this paper, the experimental characterization, finite element simulation and phase diagram calculation of the test steel were carried out, then the microstructure and properties of different parts of Nb microalloying of bridge cable wire rods were compared and analyzed. The phase transition interval of pearlite during the water-cooling process of bridge cable wire rods is increased due to the refinement of austenite grains, and the significant increase in the end temperature of the phase transition makes the average interlamellar spacing of pearlite increase. The cooling rate of different parts of bridge cable wire rods simulated by Abaqus has little difference. At the same time, Nb microalloying effectively increases the proportion of low-angle grain boundaries, so that the overall average misorientation representing the surface defects is reduced. This helps to reduce the surface energy and increase the stability of the microstructure. Combined with the mechanical properties of microtensile rods, it is found that the grain refinement effect of Nb is greater than that of coarsening interlamellar spacing during hot rolling deformation in actual production, which makes the tensile strength at the 1/4 section increase significantly. The overall tensile strength and area shrinkage of the steel wire have also been effectively improved.

## 1. Introduction

Cold-drawn steel wire is widely used in various fields of engineering due to its excellent tensile and torsional properties [1,2,3,4]. According to the composition difference of raw material bridge cable wire rods, low-carbon wire rods can be used to produce stainless steel wire [5], medium-carbon wire rods can be used to produce dual-phase steel bars and wheel wires [6], and high-carbon wire rods can be used to produce steel wire for bridge cables [7]. The improvement in the tensile strength of steel wire for ultra-high strength bridge cables will bring great economic benefits, so many researchers seek various ways to break through the strength level of existing products.

The quality control of bridge cable steel in industrial production involves many aspects, such as composition design, the heat treatment process and the deformation process. Increasing the carbon content will increase the tensile strength while reducing the plasticity, which is due to the formation of a Cottrell atmosphere [8]. This will cause the solute carbon atoms to be attracted by dislocations and affect the mechanical properties of the metal. Water cooling has a larger degree of undercooling than air cooling to obtain fine pearlite interlamellar spacing [9]. Hot rolling can fully refine the original austenite grains by recrystallization [10]. As an important microalloying element in steel, Nb has a large research foundation [11,12,13,14,15,16,17,18,19]. Especially in low-carbon steel, the grain refinement, precipitation strengthening and solid-solution strengthening have significantly improved the mechanical properties and processing properties of steel. At the same time, the Nb microalloying effect is also obvious in high-carbon steel [20]. However, in pearlitic steels with high carbon content, Nb microalloying has never been proven to have high interest. The primary function of Nb microalloying in medium- and high-carbon long products is to increase tempering stability and postpone pearlite transition, enhancing the potential of pearlite refining [21]. High-carbon steels will exhibit axial line carbon segregation in continuous casting billets due to the subsequent cementite precipitation. This has an impact on the steels’ mechanical characteristics and microstructure homogeneity [22]. It will also change the carbon equivalent, making the high-carbon component design deviate from the original purpose. The solid solution of Nb increases the carbon equivalent, but the combination of Nb and carbon causes the precipitation of a large number of Nb carbides, which lowers the carbon equivalent and helps to reduce the amount of martensite produced during cooling and the possibility of brittle fractures. Therefore, this study carried out a detailed comparative analysis of Nb microalloying of high-carbon bridge cable wire rods on the production line, in order to give more meaningful conclusions. It provides parameter guidance for the industrial production of bridge cable steel.

## 2. Materials and Methods

**Figure 1:**
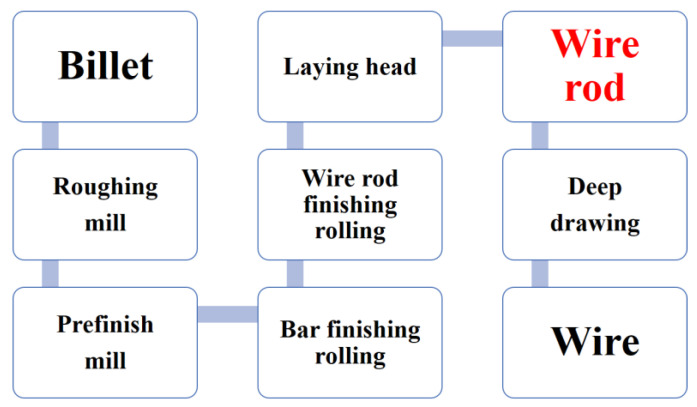
The wire steel production process.

The key processing route of hotrolled wire rods required to produce bridge cable steel is shown in Figure 1. The test steel is a bridge steel wire rod with a diameter of 14 mm, produced in the workshop as an industrial test for a typical bridge cable wire rod production line. The rolling temperature at the start was 1125–1135 °C, the rolling temperature at the end was 910–940 °C and the spinning temperature was around 900 °C. During the rolling of wire rods, 25 passes to 30 passes are taken in a continuous rolling mill. Then, the whole pearlite was obtained by Stelmor controlled cooling technology (a cooling speed of 4–10 °C/s). After 9 passes of the drawing process, the wire rod is prepared into a 7-mm-diameter steel wire. The average reduction per pass can range from 5% to 15%. The chemical compositions of the samples are presented in Table 1. A total of 0.025 wt.% Nb was added to study the effect of Nb on the bridge cable wire rod based on the previous study [18]; thus, the Nb-free sample (referred as steel A) and the Nb-bearing sample (referred as steel B) were the main specimens in this paper.

**Figure 2:**
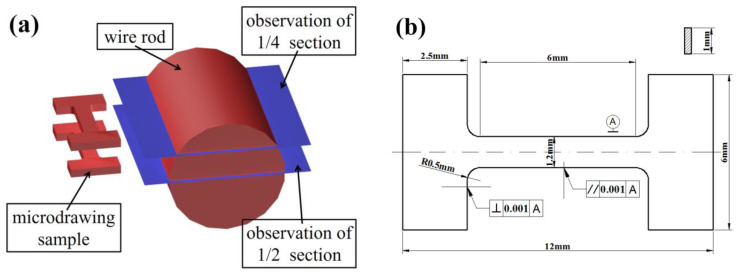
Sampling diagram of the test steel: (**a**) Sampling section position for the characterization test and microtensile test, (**b**) Schematic diagram of the microtensile sample.

**Figure 3:**
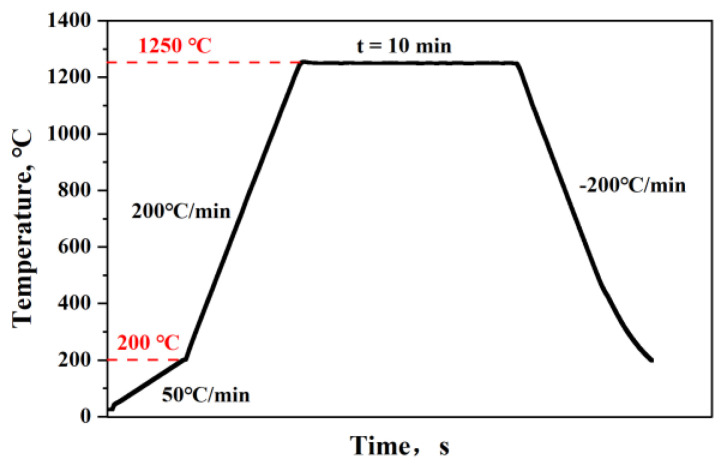
High-temperature laser-scanning confocal test process flow chart.

The bridge cable wire rod was machined to tensile test specimens with a total length of 500 mm and gauge length of 350 mm based on GB/T 228-2010. Furthermore, the 1/4 and 1/2 sections of the bridge cable wire rod were cut for experimental observation, and microtensile samples were prepared in the same section for mechanical properties tests. The sampling diagram and the microtensile size diagram are shown in Figure 2. After grinding and mechanical polishing, the samples were etched with 4% nital for 5–10 s. A scanning electron microscope was used to examine the microstructure (SEM, Quanta FEG 450, FEI Company, Hillsboro, OR, USA). The crystal orientation was observed using electron backscattered diffraction software (EBSD, Oxford Instruments/TSL OIM 7.2, Abingdon, UK). A total of 300 nm was chosen as the step size, and 20 kV was the accelerating voltage. Channel 5 software was used to process the EBSD data. Additionally, with a high-temperature laser-scanning confocal microscope, in situ observations were made to analyze the austenite transition (HT-LSCM). The process flow chart for the two composition samples is shown in Figure 3. The heating process is to simulate the production process, the holding temperature is to ensure the complete dissolution of the Nb element and the holding time is to highlight the contrast of the Nb element.

## 3. Results

### 3.1. Microstructure Characterizations

**Figure 4:**
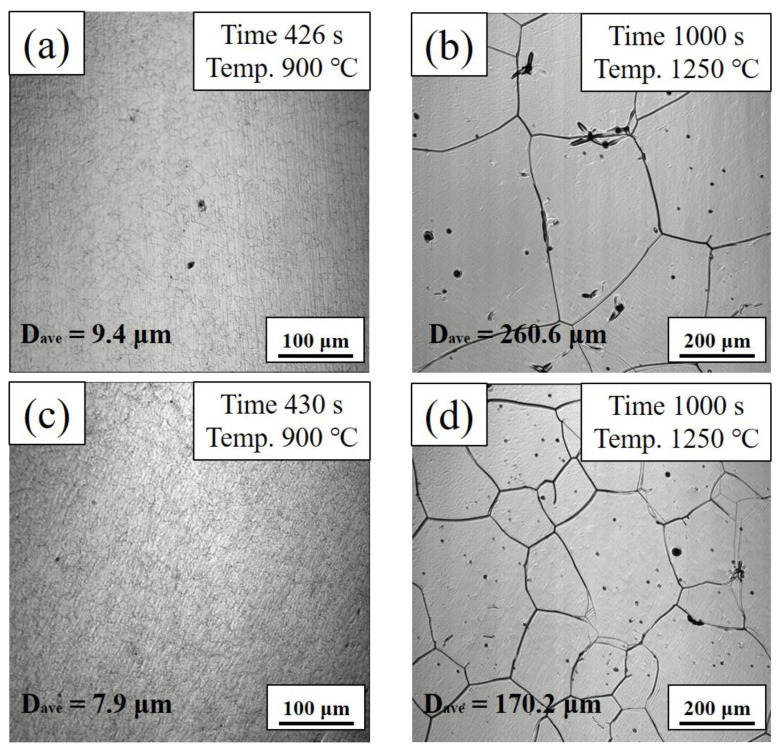
In situ observation of the austenite formation and growth process in tested steel: (**a**,**b**) steel A, (**c**,**d**) steel B.

The pearlite structure in the bridge cable wire rods has been fully dissolved when the temperature is raised to 900 °C, and the generated austenite grains are close to the hot rolling condition. The original austenite particle size (D_ave_) is recorded in the lower left corner of the image by counting the number of grains per unit area. It was 9.4 μm (Figure 4a), 260.6 μm (Figure 4b), 7.9 μm (Figure 4c) and 170.2 μm (Figure 4d). When the sample was heated to 1250 °C and held for about 400 s, the austenite grain size tends to be stable. The image of the key moment of the two samples is shown in Figure 4.

**Figure 5:**
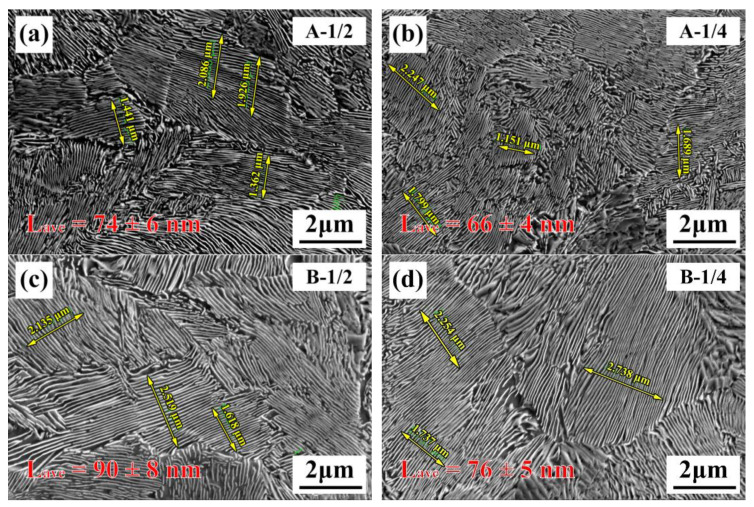
SEM images of the hot-rolled pearlite bridge cable wire rod: (**a**,**b**) steel A, (**c**,**d**) steel B.

The different sections of the test steel are all of a pearlite structure (Figure 5). The average value of pearlite interlamellar spacing (L_ave_) at each interface was calculated by the intercept method. It was 74 ± 6 nm (Figure 5a), 66 ± 4 nm (Figure 5b), 90 ± 8 nm (Figure 5c) and 76 ± 5 nm (Figure 5d).

**Figure 6:**
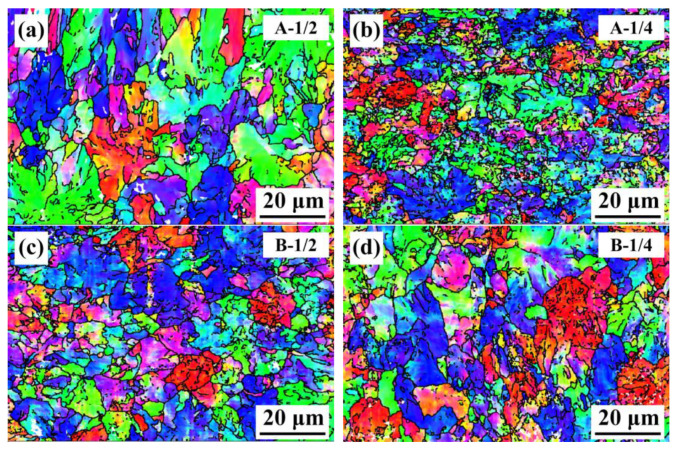
Inverse pole figures observed by EBSD: (**a**,**b**) steel A, (**c**,**d**) steel B.

Figure 6 displays the inverted pole picture of the samples, in which the grain boundaries were defined by misorientation larger than 15°. In both steel A and steel B, the microstructure is coarser in the 1/2 section as compared to the ¼ section. On the ¼ section, the grains of A are finer than those of B.

**Figure 7:**
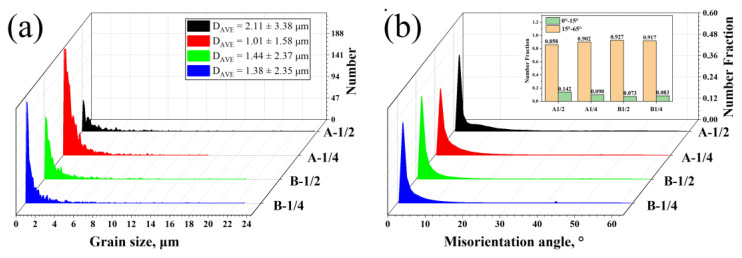
EBSD data processed by Channel 5: (**a**) average grain size and (**b**) distribution of misorientation angle.

The pearlite colony sizes and pearlite colony boundaries of the samples’ varied size angles are displayed in Figure 7a. Figure 7b shows the high- and low-angle grain boundary (H/LAGB) misorientation of the sample, and the calculations are carried out with 15° as the boundary.

### 3.2. Mechanical Properties

The microtensile mechanical properties of different parts of two samples are shown in Table 2. The tensile strength at the 1/2 section had little difference, and it increased from 1370 ± 11.5 MPa to 1405 ± 12.9 MPa at the 1/4 section. The direct tensile test results for wire rods are shown in Table 3. It can be observed that the tensile strength and reduction in the area of the steel wire are significantly improved by Nb microalloying. However, due to the inhomogeneity caused by the hot rolling process, it is necessary to characterize and analyze samples from different intercepted parts.

## 4. Discussion

### 4.1. Effect of Nb on Austenite Grains

**Figure 8:**
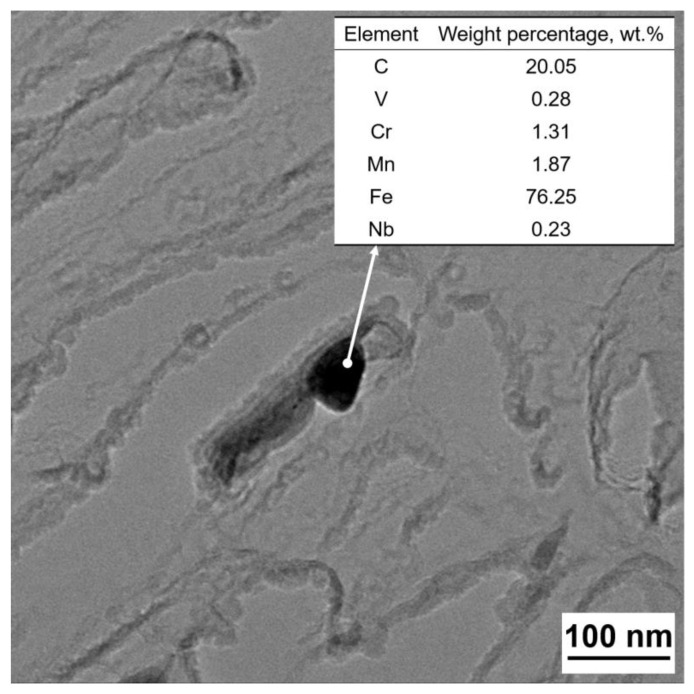
TEM image of Nb-bearing precipitate and corresponding EDS result.

Pearlite interlamellar spacing and colony size have the greatest influence on the mechanical characteristics of pearlitic steel [23]. It can be seen from Figure 4b,d that Nb microalloying has a significant inhibitory effect on the growth of austenite grains. The austenite grain sizes are 260.6 μm and 170.2 μm, respectively. Figure 4a,c also shows that the original austenite grain size after hot rolling was refined from 9.4 μm to 7.9 μm with Nb microalloying. According to our previous work [7], Nb microalloying exists in the form of solid-solution and precipitation even in high-carbon steel. At the same time, in the equilibrium state, the dissolution process of Nb was almost completed at a temperature above 1000 °C [18]. These precipitated Nb carbides play a role in pinning grain boundaries during austenite growth, as shown in Figure 8. In order to analyze the existence form of Nb in wire rod steels, we calculated the solubility product of NbC in alloy steel [24].

(1) $$\log\left[\mathrm{Nb}\right]\left[C\right]=3.04-\frac{7290}{T}$$

At a temperature of 900 °C, the mass fraction of Nb in solid solution is 0.0068 wt.%. This value is less than the addition amount of Nb 0.025 wt.%. However, Nb can be completely dissolved in austenite at the temperature of 1250 °C. Compared with Figure 4a,b, it can be found that the austenite grain size at the end of hot rolling is very small, which is due to the fact that the recrystallization process can fully refine the austenite grains [25]. Although Nb microalloys will hinder the recrystallization process, their refinement together determines the refinement of austenite grains in the spinning stage of bridge cable wire rod production. The cooling process will also be simulated based on the original austenite grain sizes of 9.4 μm and 7.9 μm.

### 4.2. Phase Transformation in the Cooling Process of Bridge Cable Wire Rods

**Figure 9:**
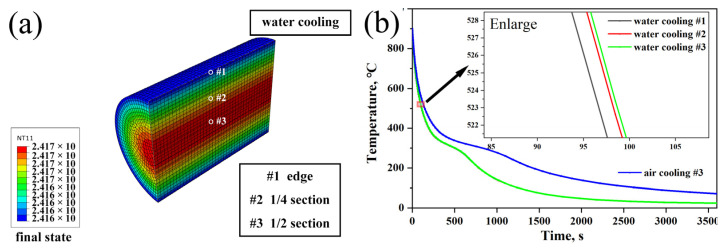
(**a**) Abaqus software simulation of bridge cable wire rod water-cooling final state diagram; (**b**) Temperature–time curves at the edge, 1/4 section and 1/2 section of the bridge cable wire rods under water and air cooling.

It is well known that the microalloying composition affects the phase transition point of steel [26]. However, the phase transition point is also affected by the austenite particle size and cooling rate [27]. To be more realistic, Abaqus/CAE 2021 was utilized to model the cooling process of the bridge cable wire rods from a spinning temperature of 900 °C to a room temperature of 20 °C. The relationship of the density, thermal conductivity and specific heat capacity of the test steel to the temperature was calculated by JMatPro 7.0.0 and substituted into the Abaqus software to simulate the cooling process when the boundary conditions were air and water. Finally, we intercepted the temperature evolution process of the three nodes shown in Figure 9a for comparison. It was discovered that the cooling rate of the 1/2 and 1/4 sections inside the bridge cable wire rod is nearly identical, with the exception that the cooling rate of surface #1 is slightly larger than that of the bridge cable wire rods. When the temperature is close to the phase transition range, the edge is about 5 °C lower than the 1/4 section, and the 1/4 section is about 1 °C lower than the 1/2 section. Compared with the water-cooling process and air-cooling process, the cooling rate of the water-cooling process obviously improved, as shown in Figure 9b. Since the water-cooling temperature at the 1/4 section and 1/2 section of the whole cooling process is only about 1 °C, the water-cooling speed is not the main reason for the difference in the performance of the experimental specimen.

**Figure 10:**
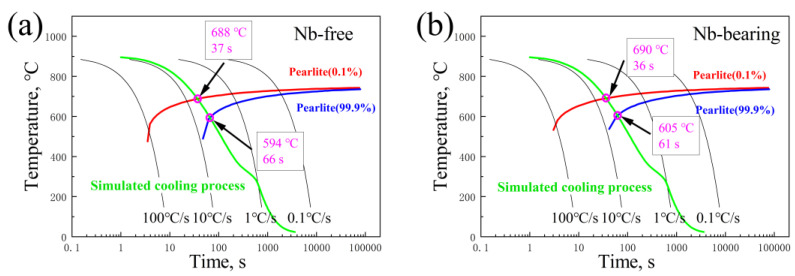
Phase transformation points on CCT curves of test steels for cooling process simulated by JMatPro: (**a**) Nb-free samples, (**b**) Nb-bearing samples.

The bridge cable wire rods’ cooling process relationship is well understood. The continuous cooling phase transition interval of the test steel with various composition and original austenite grain sizes is then calculated using JMatPro, as shown in Figure 10. The water-cooled temperature–time relationship curve obtained by simulation is used here. Despite the fact that Nb tends to shift the C curve to the right [28], the Nb microalloy still increases the temperature of pearlite transformation in the name of refining the original austenite grains. This demonstrates that the influence of the original austenite grain size on the pearlite transformation interval is dominant. The end temperature of the pearlite phase transition significantly increased from 594 °C to 605 °C. It is worth noting that the influence of microalloying on pearlite formation is much greater than the effect of physical simulation calculations [29], so the actual transition temperature of pearlite will increase more. This will greatly affect the supercooling degree of pearlite phase transitions. The pearlite interlamellar spacing obtained in the Nb-containing test steel in Figure 5 is coarser than that in the Nb-free test steel due to this reason. Moreover, as revealed by Liu et al., the non-equilibrium distribution of Nb microalloys between cementite and ferrite refines the pearlite interlamellar spacing to some extent [19]. In addition, due to the influence of niobium carbides and the low solubilization temperature of Nb, the concentration of Nb is limited in austenite solution, reducing the temperature of pearlite formation, with a consequent reduction in its interlamellar spacing, resulting in an increase in mechanical and wear resistance. While Nb microalloys can prevent the carbon diffusion during the formation of pearlite lamellae, the change in the equilibrium transition temperature will lower the undercooling degree to a reduced extent; thus, the pearlite interlamellar spacing coarsened during continuous cooling. The difference of pearlite interlamellar spacing in different sections of the same steel is also obvious. This may be due to the different deformation degree of hot rolling in different regions.

The pearlite interlamellar spacing is not only affected by the soft impact caused by the diffusion of C and alloy particles [30], but also by thermal fluidization and recalescence [31]. The degree of undercooling is the main factor in the conventional empirical formula, and its impact is particularly clear. We used Zener’s semi-empirical formula [32]:

(2) $$\lambda =\frac{\alpha \sigma T_{E}}{\Delta H_{V}(T_{E}-T)}$$

The interlamellar spacing of pearlite *λ* and the constant 4 in the formula indicate that the pace of pearlite development is governed by the volume diffusion of carbon in austenite. *σ* (=0.40~0.70 J/m^2^) [33] is the stated ferrite/cementite interfacial energy values. Use undercooling Δ*T *for the equilibrium transition temperature of pearlite and Δ*H_V_* (=607 MJ/m^3^) [34] for the change in enthalpy per unit volume associated with pearlite transformation. Formula (2) can be simplified as follows if the specific interfacial energy and molar volume of test steel do not vary much after adding Nb:

(3) $$\lambda =\frac{KT_{E}}{\Delta T}$$

*K* is a constant in the formula. The undercooling range at the end point of the transformation of two different test steels is considered, and the real interlamellar spacing is turned into the apparent interlamellar spacing. The obtained ranges are: steel A, 34.6–108.5 nm; steel B, 37.6–113.7 nm. In comparison to Figure 5, the experimental data fall exactly within the range calculated by the supercooling factor. However, the mechanisms producing the variation in lamellar spacing between the 1/2 and 1/4 sections must be investigated further.

### 4.3. Surface Defects in Pearlitic Steel

The rolling action had stored some deformation energy, some of which would be consumed by the austenite recrystallization process. When the crystals undergo the pearlite transition, the leftover deformation energy causes the production of dislocations and point defects [35]. The dislocations gradually intersect to generate dislocation walls and subgrain boundaries as more dislocations build up in the crystal. In the event that the subgrain borders keep absorbing dislocations, they will eventually change into HAGBs. This series of microstructure defects has an impact on the mechanical properties of steel [36]. For example, the initial austenite grain boundary and the precipitated Nb carbides will act as nucleation particles to induce the nucleation and growth of pearlite during the pearlite phase transformation process [37]. It is also difficult to quantify the difference in interface misorientation between pearlite clusters and pearlite fields. As a result, the conventional statistical technique of a grain size with a 15° misorientation as the grain boundary is not always applicable to pearlite. The grain size of pearlite measured by this method has no discernible regularity, as illustrated in Figure 7a. In Figure 7b, all the surface defects represented by the orientation difference of 0–65° are taken into account, and the contribution of surface defects in steel is represented by calculating the average orientation difference.

Similar to the calculation of the 0–5° average misorientation by kernel average misorientation (KAM) and the geometrically necessary dislocation density, the overall average misorientation (OAM) can be calculated using the following formula [38].

(4) $$\theta =OAM_{\mathrm{ave}}=\exp\left[\frac{1}{N}\overset{i}{\underset{1}{\sum }}\ln OAM_{L,i}\right]$$

In Figure 7b, the distribution of *OAM* was calculated for the four samples. *OAM_L,i_* represents the local *OAM* value at point *i*, and *N* represents the number of points in the test region. The average misorientations representing the surface defects in the samples are A-1/2: 8.03°, A-1/4: 6.53°, B-1/2: 5.92° and B-1/4: 5.46°. As shown in Table 2, the mechanical properties of the identical bridge cable wire rods are greatly improved by lowering the OAM at the 1/4 section and refining the interlamellar spacing. As a result, we have reason to suppose that the smaller the average misorientation of the representative surface defects in pearlite, the finer the grain size [39].

The mechanical properties of the 1/2 section of the bridge cable wire rods after Nb microalloying are comparable when the lamellar spacing, average misorientation and tensile mechanical properties of the four samples are considered in Table 2. In this situation, Nb microalloying effectively improves the surface defects of pearlite while also increasing the phase-transition end temperature to thicken the pearlite lamellae. The mechanical properties of the two types of bridge cable wire rods are better at the 1/4 section than at the 1/2 section, demonstrating that surface defects have a greater impact on pearlite’s mechanical properties. The smaller the overall average misorientation angle, the lower the surface energy and the more stable the pearlite structure. The effect of Nb microalloying to refine the original austenite by pinning grain boundaries is well recognized; however, it is crucial to note that this method also considerably increases the proportion of LAGBs. The interphase precipitation of Nb microalloys is to blame for this [40]. The precipitates are a result of the repeated nucleation of alloy carbides on migrating austenite(γ)/ferrite(α) interfaces during the pearlite transformation. This will severely restrict the movement of dislocations during plastic deformation [41,42,43]. When the mechanical properties of the 1/4 sections of the two bridge cable wire rods are compared, Nb microalloying enhances mechanical properties more. The previous simulation reveals that there is no discernible variation in the cooling rate between the two portions, but the interlamellar spacing is greatly refined. This is because the two portions deformed differently during the hot rolling process. After hot rolling, the microstructure at the 1/4 section stores higher deformation energy. These energies will not only encourage pearlite nucleation and growth, but will also promote Nb carbide interphase precipitation, increasing the number and fraction of LAGBs. Ultimately, the performance of the steel wire is effectively improved.

## 5. Conclusions

The microstructure of different Nb microalloyed bridge cable wire rod cross-sections was compared. Because of the complexity of the real production process, the contribution of Nb in high-carbon steel is the aggregate of various elements. The following conclusions can be drawn:

(1) The Nb microalloying in the tested steel refined the austenite grains. The calculated phase diagram shows that the start temperature of the pearlite transition increased by 2 °C and the end temperature increased by 9 °C. This leads interlamellar spacing in different areas of pearlite to coarsen.

(2) The simulation shows that the temperature at the edge is about 5 °C lower than the temperature at the 1/4 section, and the temperature at the 1/4 section is about 1 °C lower than the temperature at the 1/2 section. It can be concluded that the main reason for the difference in structural properties of different cross-sections of steel wire is the uneven plastic deformation of the drawing process.

(3) Because of the difference in deformation energy storage accumulated during the hot rolling process and Nb microalloying, the proportion of LAGB increases substantially after Nb microalloying. The overall average misorientation of the four test samples is as follows: A-1/2: 8.03°, A-1/4: 6.53°, B-1/2: 5.92° and B-1/4: 5.46°. A decrease in this value means an increase in the tensile strength at different cross-sections of the steel wire.

(4) According to the experimental data of interlamellar spacing and surface defects representing grain size, it is thought that the precipitation of Nb microalloying during hot rolling hinders the migration of the low-angle boundary in the real production process, and the improvement of tensile strength is greater than its coarsening effect on lamellar spacing.

## Figures and Tables

**Table 1:** Chemical compositions of the samples (wt.%).

Steel	C	Si	Mn	Cr	P	S	V	Nb	N	Fe
A	0.93	0.85	0.78	0.26	0.012	0.001	0.04	-	0.0035	Bal.
B	0.93	0.85	0.78	0.26	0.009	0.002	0.04	0.025	0.0036	Bal.

**Table 2:** Average microtensile properties and error of samples (MPa).

	Tensile Strength	Elongation, %
A-1/2	1300 ± 10	17 ± 1
A-1/4	1370 ± 12	14 ± 2
B-1/2	1302 ± 10	15 ± 1
B-1/4	1405 ± 13	13 ± 2

**Table 3:** Direct tensile test results of bridge cable wire rods.

Specimen	Tensile Strength (MPa)	Reduction of Area (%)
A	1402 ± 15	21 ± 2
B	1446 ± 10	38 ± 1

## Data Availability

Not applicable.
